# The Optimal Treatment for Resectable Esophageal Cancer: A Network Meta-Analysis of 6168 Patients

**DOI:** 10.3389/fonc.2021.628706

**Published:** 2021-03-10

**Authors:** Meng Yuan, Yongxing Bao, Zeliang Ma, Yu Men, Yang Wang, Zhouguang Hui

**Affiliations:** ^1^ Department of Radiation Oncology, National Cancer Center/National Clinical Research Center for Cancer/Cancer Hospital, Chinese Academy of Medical Sciences and Peking Union Medical College, Beijing, China; ^2^ Department of VIP Medical Services, National Cancer Center/National Clinical Research Center for Cancer/Cancer Hospital, Chinese Academy of Medical Sciences and Peking Union Medical College, Beijing, China; ^3^ Institute of Basic Medical Sciences, Chinese Academy of Medical Sciences and Peking Union Medical College, Beijing, China

**Keywords:** adjuvant, neoadjuvant, radiotherapy, chemotherapy, chemoradiotherapy, esophageal cancer, network meta-analysis

## Abstract

The optimal treatment for resectable esophageal cancer remains unclear. This network meta-analysis compares the efficacy of different treatments. PubMed, Embase, and the Cochrane library were systematically screened. Randomized controlled trials comparing the efficacy of different treatments for resectable esophageal cancer were included. Hazard ratios (HR) for overall survival (OS), progression-free survival, or disease-free survival, and odds ratios for locoregional recurrence and distant metastasis rates were identified as the measurements of efficacy. A Bayesian network meta-analysis was performed. In this study, 26 studies were included. Patients received either surgery alone; neoadjuvant chemotherapy (CT), neoadjuvant radiotherapy (RT), or neoadjuvant chemoradiotherapy (CRT) followed by surgery; or surgery followed by adjuvant CT, adjuvant RT, or adjuvant CRT. Neoadjuvant CRT followed by surgery (pooled HR = 0.76, 95% credible interval: 0.67–0.85) and neoadjuvant CT followed by surgery compared with surgery alone were the only two showing statistically confident improvement on OS. Ranking analysis showed that neoadjuvant CRT with surgery was likely to be the best option in terms of efficacy. Therefore, for patients with resectable esophageal cancer, neoadjuvant CRT with surgery is the optimal treatment. Future studies should focus on the optimization of neoadjuvant CRT regimens.

## Introduction

Esophageal cancer is the sixth leading cause of cancer-associated mortality worldwide due to its highly aggressive nature and poor prognosis (1). The long-term outcomes of esophageal cancer patients remain poor with 5-year survival rates of 15%–35% even in cases of resectable cancers (2). With the high relapse rate reaching up to 43.3%–50.0% (3–5), surgery alone exhibits limited therapeutic effect. Therefore, neoadjuvant and adjuvant treatments, including chemotherapy (CT), radiotherapy (RT), and chemoradiotherapy (CRT), have been administered to improve survival of patients with resectable esophageal cancer. For the last decades, numerous prospective randomized controlled trials (RCT) have tried to evaluate the efficacy of these neoadjuvant or adjuvant treatments. However, results were not consistent across trials. Recently, several phase III trials have brought notable outcomes, such as the results from NEOCRTEC5010 and long-term results of the CROSS study, demonstrating that neoadjuvant CRT significantly increased overall survival (OS) compared with surgery alone (6, 7). Long-term results of the POET study suggest a survival benefit for preoperative CRT compared with preoperative CT (8), whereas results from the NeoRes I trial do not support unselected addition of RT to neoadjuvant CT as a standard of care in patients with resectable esophageal cancer (9). Still, the optimal sequence and combination strategies of CT, RT, or CRT among all of these treatments are unclear.

Traditional pair-wise meta-analysis cannot integrate all the evidence of different treatments at the same time, and the network meta-analysis offers the opportunity to perform both direct and indirect treatment comparisons among randomized studies simultaneously. Due to the lack of conclusive evidence and the difficulties in the conduction of adequately designed multidimensional clinical trials, this systematic review and network meta-analysis evaluates the relative efficacy of neoadjuvant or adjuvant CT, RT, or CRT and surgery or surgery alone for patients with resectable esophageal cancer in RCTs.

## Methods

This network meta-analysis was performed following the preferred reporting items for systematic reviews and meta-analyses (PRISMA) extension statement for network meta-analysis (**Supplementary Table 1**). The protocol was registered in the Prospective Register of Systematic Reviews (PROSPERO CRD42020168448).

### Data Sources and Searches

PubMed, Embase, and the Cochrane Central Register of Controlled Trials were systematically screened from inception to June 2020 using a combination of the main search terms “neoadjuvant (preoperative) therapy” and “adjuvant (postoperative) therapy” and “chemotherapy or radiotherapy (radiation, irradiation) or chemoradiotherapy (chemoradiation, chemoirradiation, radiochemotherapy)” and “esophageal (oesophageal, esophagus, oesophagus) or esophagogastric (oesophagogastric, gastric esophageal, gastroesophageal) junction cancer (carcinoma, neoplasm, tumor)” within the restriction of “randomized controlled trial” and “English language” (detailed search strategy in **Supplementary Materials**). Manual searches from previous meta-analyses were also performed.

### Study Selection

We included published prospective RCTs that met the following criteria:

1. Phase II/III RCTs and RCTs with study population larger than 50.
2. Trials that enrolled patients with histologically confirmed adenocarcinoma (AC) or squamous cell carcinoma (SCC) of the esophagus or gastro-esophageal junction and were fit for potentially curative surgery.
3. Trials that compared any two or more different treatments (neoadjuvant CT, RT, or CRT with surgery; surgery with adjuvant CT, RT, or CRT; and surgery alone) in patients with resectable esophageal cancer.
4. For trials comparing neoadjuvant (C)RT or adjuvant (C)RT with other treatments, the prescription dose of RT set in the protocol should be more than 20 Gy.
5. Trials that reported at least one of the following clinical outcome measures: OS, defined as the time from randomization until the last follow-up or death; progression-free survival (PFS), defined as the time from randomization to first progression (locoregional or distant) or the last follow-up or death; disease-free survival (DFS), defined as the time from R0 resection to disease recurrence or the last follow-up or death; the number (or the rates) of treatment failures due to locoregional recurrence and distant metastasis.

Only full-text articles published in English were included. If multiple publications of the same trial were retrieved, the most recent and informative publication was included. Studies also enrolling patients with gastric cancer were excluded for potential bias due to the heterogeneity of patient characteristics.

### Data Extraction

Two authors were responsible for screening the titles and abstracts of the retrieved references. The full texts of the included studies were assessed based on the aforementioned criteria. Any discrepancies were resolved by consensus and arbitration by a panel of senior authors. Data on trial details (study ID, first author, publication year, number of patients, baseline characteristics of study population), treatments, and outcomes (in particular, hazard ratios (HR) with their 95% confidence intervals (CI) for OS, PFS, or DFS and the number of patients experiencing locoregional relapse or distant metastasis or rates of locoregional recurrence and distant metastasis) were extracted.

### Risk of Bias Assessment

The quality of each eligible study was evaluated by the revised Cochrane risk-of-bias tool for randomized trials (RoB 2) (version of August 22, 2019). The entire scale comprises the following domains: randomization process, deviations from intended interventions, missing outcome data, measurement of the outcome, selection of the reported result, and overall bias. According to the detailed guidance of RoB 2, each domain could be judged as any of the three levels: low, high, or unclear risk of bias. The associated data were extracted and assessed using predefined fields.

Publication bias was assessed by visual inspection of the funnel plot.

### Data Synthesis and Statistical Analysis

The HR for OS was proposed as the primary interest of efficacy, which takes into account the timing and censoring of survival status. To obtain the HR and its standard error, three approaches were applied (1): For studies that reported the summary statistics, HR was directly collected, and standard error was calculated from CI (2). In the absence of summary statistics, some studies published the Kaplan–Meier curve with at-risk table. Using a method proposed by Tierney (10), survival rate and number at risk were extracted from a plot based on the time intervals divided schematically. Number of survivals, deaths, and censors were estimated for every time interval. HR and standard error were calculated by combining all time intervals (3). For studies that published the Kaplan–Meier curve with the follow-up information instead of at-risk table, similar steps were applied, whereas the estimate of censor was approximated based on a linear pattern. The same approaches were applied to obtain other time-to-event outcomes, such as PFS and DFS. Locoregional recurrence and distant metastasis rate were identified as supportive measures for OS. The odds ratio (OR) and its standard error was calculated directly from the number of events and sample size without any adjustment in term of missingness.

Bayesian network meta-analysis (NMA) was carried out to synthesize all therapeutic options within a mixed treatment comparison framework using R 3.6.0. A random effects model was prioritized to address the study-specific effects that were a component of overarching distribution. To be objective, uninformative prior distribution was given to all parameters. The node-split method was used to assess inconsistency.

The estimates of relative effects and 95% credible intervals (CrI) were reported. In addition, the surface under the cumulative ranking (SUCRA) scores were calculated as well.

## Results

### Characteristics of the Included Studies

**Figure 1:**
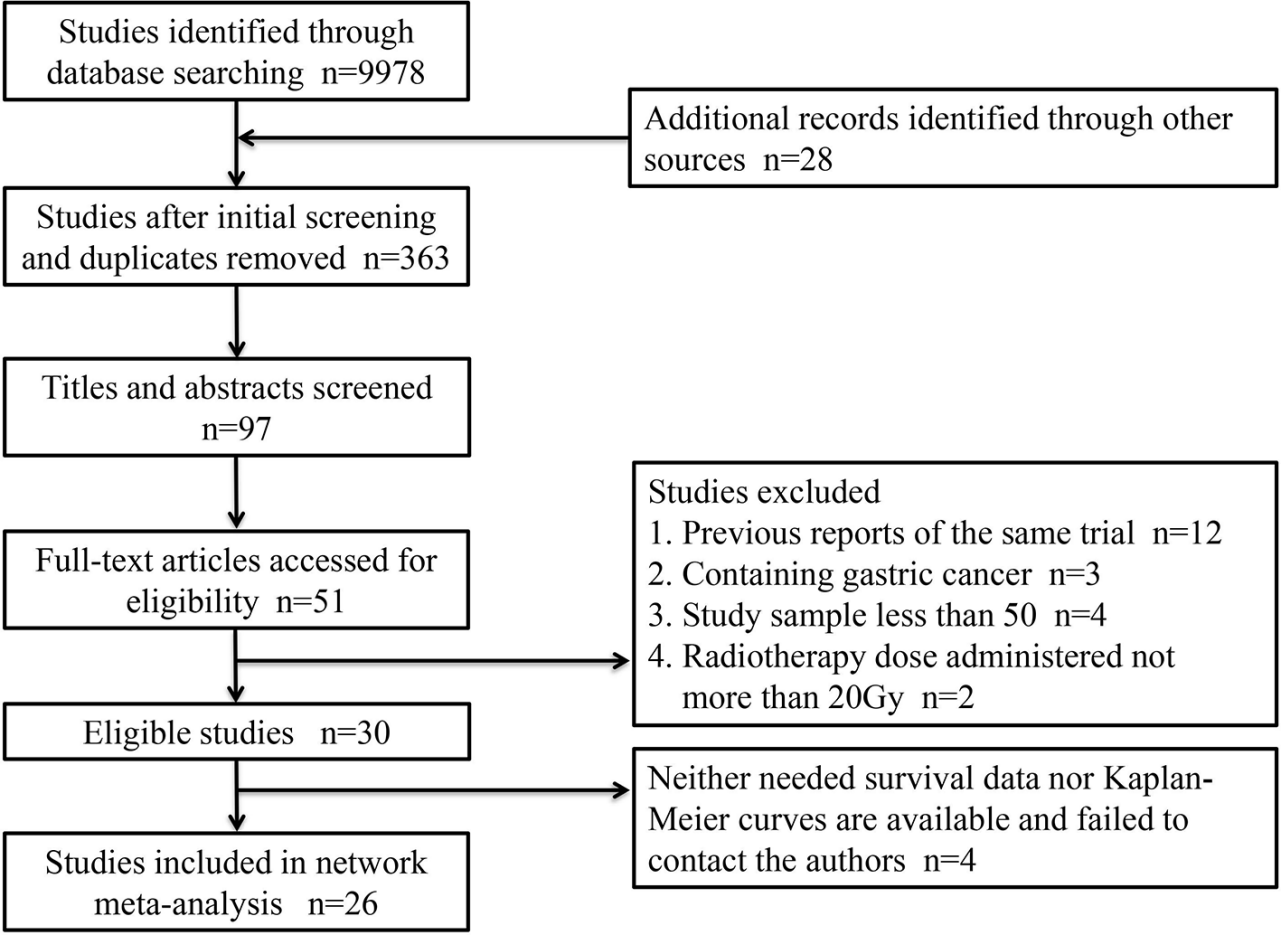
Selection flow chart.

**Figure 2:**
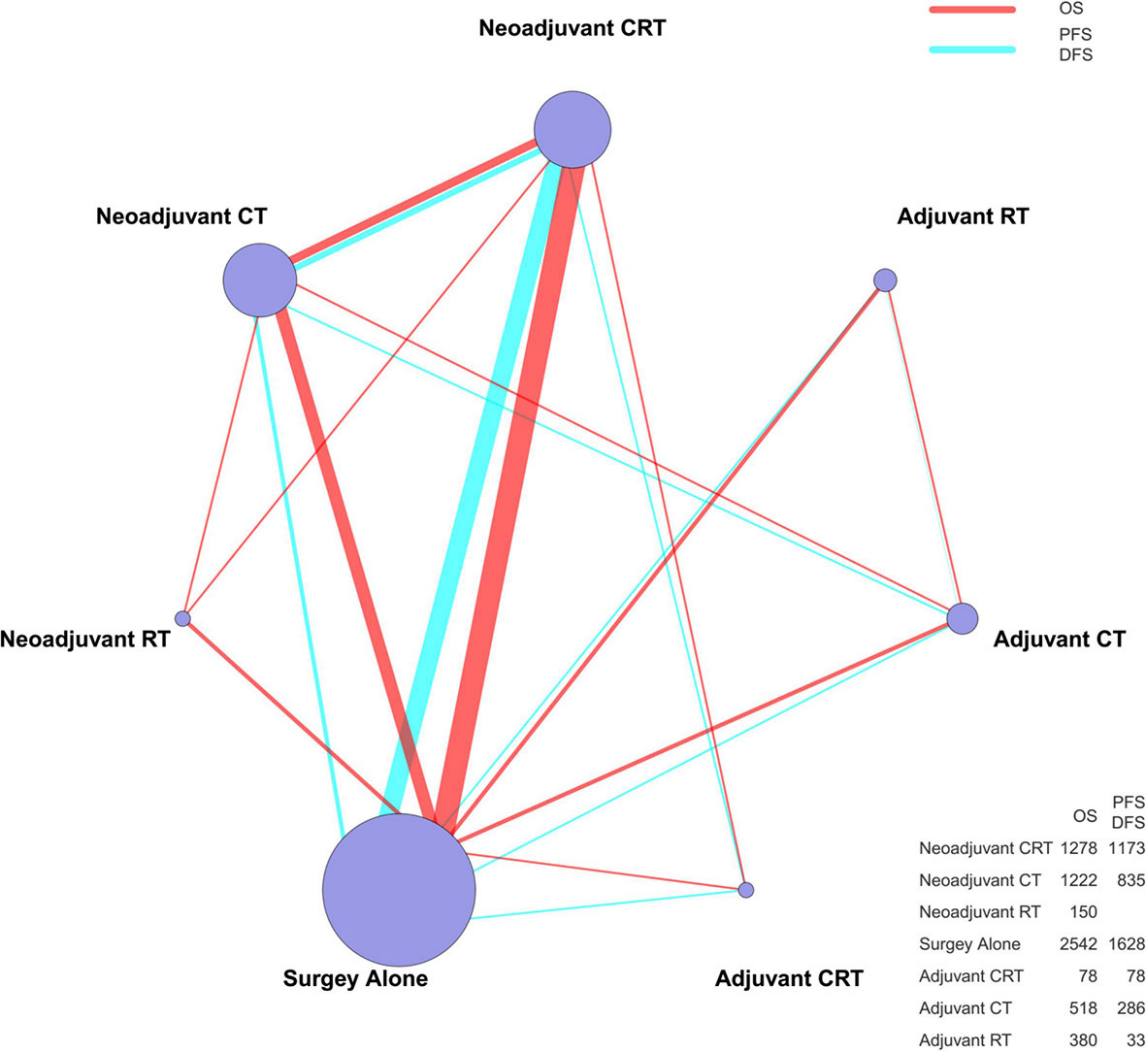
The mixed treatment comparison framework of OS and PFS/DFS in patients with resectable esophageal cancer. The node size is proportional to the total number of patients receiving a specific treatment. Each line represents a type of head-to-head comparison. The width of lines is proportional to the number of trials comparing the connected treatments. CT, chemotherapy; RT, radiotherapy; CRT, chemoradiotherapy; OS, overall survival; PFS, progression-free survival; DFS, disease-free survival.

A total of 26 articles were eligible for the NMA (6–9, 11–32). **Figure 1** outlines the selection process flow. In the included RCTs, 6168 patients were included to receive either surgery alone (*n* = 2542); neoadjuvant CT (*n* = 1222), neoadjuvant RT (*n* = 150), or neoadjuvant CRT (*n* = 1278) followed by surgery; or surgery followed by adjuvant CT (*n* = 518), adjuvant RT (*n* = 380), or adjuvant CRT (*n* = 78). The main characteristics of all studies are reported in **Table 1**. The mixed treatment comparison framework of OS together with PFS/DFS is shown in **Figure 2**.

**Table 1:** Baseline characteristics of studies included in the NMA.

Author	Year	Design	No. pts	Tumor type	Disease location	RT schedule	CT schedule
Yang et al. (7)	2018	NCRT-S vs S	451	SCC	thoracic esophagus	40Gy/2Gy/20f	two cycles vinorelbine 25mg/m^2^ d1,8 + cisplatin 75mg/m^2^ d1 q3w
von Dobeln et al. (9)	2019	NCRT-S vs NCT-S	181	AC, SCC	esophagus or GEJ	40Gy/2Gy/20f	three cycles cisplatin 100mg/m^2^ d1 + fluorouracil 750mg/m^2^/d, d1–5
Shapiro et al. (6)	2015	NCRT-S	368	AC, SCC, undifferentiated carcinoma	esophagus or GEJ	41.4Gy/1.8Gy/23f	five cycles carboplatin AUC 2mg/mL/min d1, 8, 15, 22, 29 + paclitaxel 50mg/m^2^ d1, 8, 15, 22, 29
Urba et al. (29)	2001	NCRT-S vs S	100	AC, SCC	esophasus	45Gy/1.5Gy bid	cisplatin 20mg/m^2^/d d1-5,17-21 + fluorouracil 300mg/m^2^/d d1-21 + vinblastine 1mg/m^2^/d d1-4,17-20
Stahl et al. (8)	2017	NCRT-S vs NCT-S	119	AC	GEJ	30Gy/2Gy/15f	induction chemotherapy 14 weeks: 5-fluorouracil 2g/m^2^ qw + folinic acid 500mg/m^2^ qw + cisplatin 50mg/m^2^ q2w; concurrent chemotherapy 3 weeks: cisplatin 50 mg/m^2^ d2,8 + etoposide 80mg/m^2^ d3-5
Xiao et al. (31)	2003	S-ART vs S	495	SCC	esophagus	60Gy/30f, transpositioned stomach:50Gy/25f	NA
Law et al. (23)	1997	NCT-S vs S	147	SCC	thoracic esophagus	NA	two cycles cisplatin 100mg/m^2^ d1 + fluorouracil 500mg/m^2^/d d1–5
Walsh et al. (30)	1996	NCRT-S vs S	113	AC	esophagus excluding cervical esophagus	40Gy/15f	two cycles cisplatin 75 mg/m^2^ d7 + fluorouracil 15 mg/kg/d d1–5
Zieren et al. (32)	1995	S-ART vs S	68	SCC	thoracic esophagus	55.8Gy/1.8Gy/31f	NA
Tepper et al. (28)	2008	NCRT-S	56	AC, SCC	thoracic esophagus (below20cm) or GEJ	50.4Gy/1.8Gy bid	cisplatin 100mg/m^2^/d + fluorouracil 1,000 mg/m^2^/d for 4 days on weeks 1 and 5
Mariettte et al. (26)	2014	NCRT-S vs S	195	AC, SCC	thoracic esophagus	45Gy/1.8Gy/25f	two cycles fluorouracil + cisplatin d1-5, 29-33
Lee et al. (24)	2004	NCRT-S vs S	101	SCC	thoracic esophagus	45.6Gy/1.2Gy/38f	cisplatin 60mg/m^2^ d1,22 + 5-fluorouracil 1000mg/m^2^ d2–5 (For patients with disease stable or responsive to CRT, three additional cycles of chemotherapy were given after surgery)
Lv et al. (25)	2010	NCRT-S vs S-ACRT vs S	238	SCC	thoracic esophagus	40Gy/2Gy/20f	two cycles PTX 135mg/m^2^/d + DDP 20 mg/m^2^/d d1-3, 22-24
Ancona et al. (12)	2001	NCT-S vs S	94	SCC	esophagus	NA	two cycles cisplatin 100mg/m^2^ d1, 21 + 5-fluorouracil 1000mg/m^2^ d1–5, 21-26
Nygaard et al. (27)	1992	S vs NCT-S vs NRT-S vs NCRT-S	186	SCC	located at least 21 cm from the incisor teeth, or below the 5th thoracic vertebra	35Gy/1.75Gy/20f	two cycles cisplatin 20mg/m^2^ d1-5 + bleomycin 5mg/m^2^ d1
Allum et al. (11)	2009	NCT-S vs S	802	AC, SCC, undifferentiated carcinoma	esophagus or GEJ	NA	two cycles cisplatin 80mg/m^2^ d1 + fluorouracil 1000 mg/m^2^ daily as a continuous infusion over 96 hours q3w
Boonstra et al. (16)	2011	NCT-S vs S	169	SCC	thoracic esophagus	NA	two-four cycles cisplatin 80mg/m^2^ d1, etoposide 100 mg/m^2^ d1,2, followed by etoposide 200mg/m^2^ orally d3,5 q3w
Bosset et al. (17)	1997	NCRT-S vs S	282	SCC	thoracic esophagus	37Gy/3.7Gy/10f	two cycles cisplatin 80mg/m^2^, d1-3
Burmeister et al. (18)	2005	NCRT-S vs S	256	AC, SCC	thoracic esophagus	35Gy/2.3Gy/15f	one cycle cisplatin 80mg/m^2^ d1+ fluorouracil 800mg/m^2^ d1–4
Ando et al. (14)	1997	S-ACT vs S	205	SCC	thoracic esophagus	NA	two cycles cisplatin 70mg/m^2^ + vindesine 3mg/m^2^ d1, 21
Ando et al. (15)	2012	S-ACT vs NCT-S	330	SCC	thoracic esophagus	NA	two cycles cisplatin 80mg/m^2^ d1+ 5-fluorouracil 800mg/m^2^ d1-5
Ando et al. (13)	2003	S-ACT vs S	242	SCC	thoracic esophagus	NA	two cycles cisplatin 80mg/m^2^ d1 + fluorouracil 800mg/m^2^ d1-5
Jeog (21)	1993	S-ART vs S-ACT	258	NA	cervical and thoracic esophagus	50Gy/2Gy/25f	three cycles cisplatin 50mg/m^2^ + vindesine 3mg/m^2^ d1
Kelsen et al. (22)	2007	NCT-S vs S	243	AC, SCC	esophagus or GEJ	NA	three cycles cisplatin 100mg/m^2^ d1 + 5-fluorouracil 100mg/m^2^ d1-5
Burmeister et al. (19)	2011	NCT-S vs NCRT-S	75	AC	esophagus or GEJ	35Gy/2.3Gy/15f	two cycles cisplatin 80mg/m^2^ d1 + 5-fluorouracil 1000mg/m^2^/d d1-5 q3w
Gignoux et al. (20)	1987	NRT-S vs S	208	SCC	thoracic esophagus	33Gy/3.3Gy/10f	NA

NCRT-S, neoadjuvant chemoradiotherapy followed by surgery; NCT-S, neoadjuvant chemotherapy followed by surgery; NRT-S, neoadjuvant radiotherapy followed by surgery; S, surgery; S-ACRT, surgery followed by adjuvant chemoradiotherapy; S-ACT, surgery followed by adjuvant chemotherapy; S-ART, surgery followed by adjuvant radiotherapy; SCC, squamous cell carcinoma; AC, adenocarcinoma; GEJ, gastroesophageal junction; qw, weekly; q2w, biweekly; q3w, every 3 weeks.

### Homogeneity and Transitivity

No included trials were judged as high risk of bias concerning study design (**Supplementary Figure 4**). Generally, all included trials were comparable in terms of clinical features. The assumption of transitivity was accepted. We categorized and reorganized studies according to different treatments into 7 arms to make comparisons. Among the neoadjuvant CRT arm, however, in Lee’s study (24), which aimed to compare the efficacy of neoadjuvant CRT with surgery and surgery alone for patients with disease that was stable or responsive to CRT, three additional cycles of chemotherapy were actually given after surgical resection. Besides this, in Mariette’s study (26), the patients enrolled were only those of stage I or II. Therefore, we decided to exclude these two studies in the sensitivity analysis to enhance the robustness as well as to detect the stability of the outcome.

### Inconsistency Assessment

Inconsistency between direct and indirect evidence was assessed with the node-split method. No major differences between direct and indirect evidence was detected in all these comparisons (*p* > 0.05) except for NRT-S versus NCT-S (*p* = 0.026) in the subgroup analysis of SCC for OS (**Supplementary Figure 1**).

### Results of NMA

**Figure 3:**
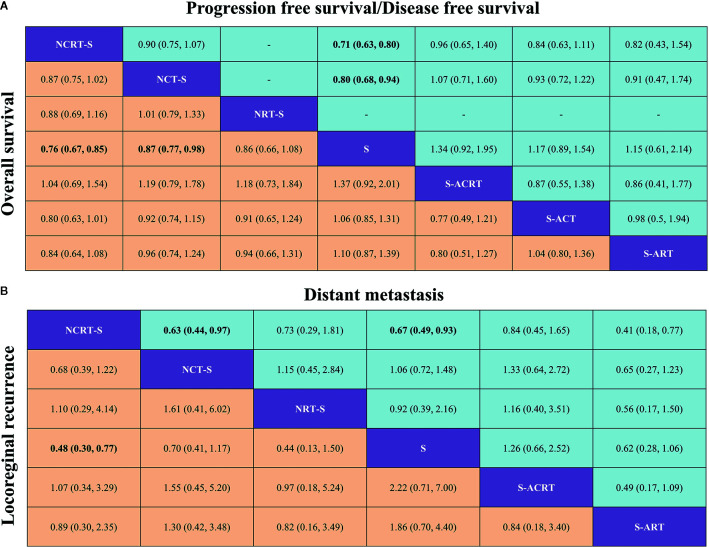
**(A)** Pooled relative effects of all treatment comparisons in the NMA. Pooled HRs (95% CrIs) for PFS/DFS (upper triangle) and OS (lower triangle). Data in each cell are HRs (95% CrIs) for the comparison of row- versus column-defining treatment for PFS/DFS or the comparison of column- versus row-defining treatment for OS. Statistically confident results are in bold. NCRT-S, neoadjuvant chemoradiotherapy followed by surgery; NCT-S, neoadjuvant CT followed by surgery; NRT-S, neoadjuvant RT followed by surgery; S, surgery; S-ACRT, surgery followed by adjuvant CRT; S-ACT, surgery followed by adjuvant CT; S-ART, surgery followed by adjuvant RT. **(B)** Pooled relative effects of all treatment comparisons in the NMA. Pooled ORs (95% CrIs) for distant metastasis (upper triangle) and locoregional recurrence (lower triangle). Data in each cell are ORs (95% CrIs) for the comparison of row- versus column-defining treatment for distant metastasis or the comparison of column- versus row-defining treatment for locoregional recurrence. Statistically confident results are in bold. NCRT-S, neoadjuvant CRT followed by surgery; NCT-S, neoadjuvant CT followed by surgery; NRT-S, neoadjuvant RT followed by surgery; S, surgery; S-ACRT, surgery followed by adjuvant CRT; S-ACT, surgery followed by adjuvant CCT; S-ART, surgery followed by adjuvant RT.

An NMA was conducted to investigate the neoadjuvant and adjuvant treatments for resectable esophageal cancer (network plot of OS and PFS/DFS, **Figure 2**; relative effects of OS and PFS/DFS, **Figure 3A**).

In terms of OS, neoadjuvant CRT followed by surgery yielded the best benefit of all the treatments when compared with surgery alone (HR = 0.76, 95% CrI: 0.67–0.85). Second to it, a substantial difference was also observed in neoadjuvant CT followed by surgery compared with surgery (HR = 0.87, 95% CrI: 0.77–0.98). Adjuvant CRT apparently demonstrated a similar impact on OS although with some level of uncertainty (HR = 0.73, 95% CrI: 0.5–1.09). Besides this, the NMA also shows a trend but not a statistically confident OS benefit of neoadjuvant RT (HR = 0.86, 95% CrI: 0.66–1.08) compared with surgery alone.

Sixteen studies reported HRs for PFS/DFS or Kaplan–Meier curves for manipulation. In terms of PFS/DFS, neoadjuvant CRT (HR = 0.71, 95% CrI: 0.63–0.80) was also associated with the most robust survival advantage across different treatment options, followed by neoadjuvant CT (HR = 0.8, 95% CrI: 0.68–0.94). Adjuvant CRT (HR = 0.75, 95% CrI: 0.51–1.08) presented nonconfident OS benefit (**Figure 3A**).

In terms of failure patterns, detailed data on recurrence were available in 16 studies. The statistically confident decrease in locoregional recurrence rate could only be observed in patients receiving neoajuvant CRT (OR = 0.48, 95% CrI: 0.30–0.77, **Figure 3B**). When it comes to distant metastasis, we found similar results that only neoajuvant CRT could reduce the distant metastasis rate compared with surgery alone with statistical confidence (OR = 0.67, 95% CrI: 0.49–0.93, **Figure 3B**).

### Subgroup Analysis by Histology Types

Subgroup analysis for SCC and AC was conducted in 19 and 9 trials, respectively. For patients with SCC, both neoadjuvant CRT and neoadjuvant CT conferred an OS advantage over surgery alone: HRs (95% CrIs) were 0.76 (0.65–0.89) and 0.81 (0.7–0.94), respectively. A trend in favor of adjuvant CRT was also found in SCC: HR (95% CrI) was 0.73 (0.49–1.08). For patients with AC, neoadjuvant CRT was apparently associated with better survival (HR = 0.79, 95% CrI: 0.59–1.04). Neoadjuvant CRT with surgery ranked first (SUCRA scores: 0.79 and 0.87) in both histology types.

### Ranking of Treatments

**Figure 4:**
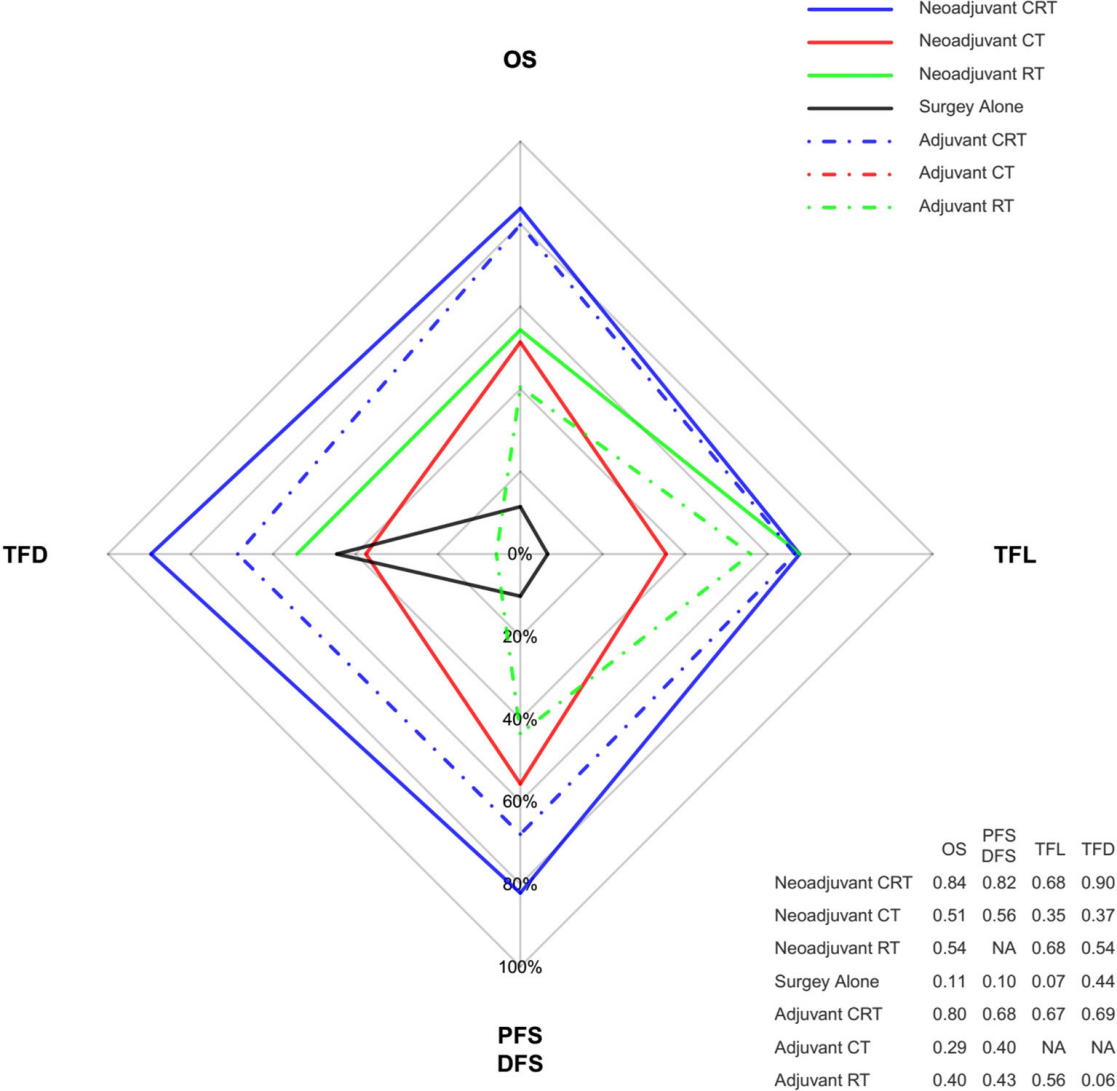
Radar map of the Bayesian ranking results of 4 different endpoints. This map indicates the probability of each comparable treatment being ranked first in terms of different endpoints. Each vertex of the rhombus represents an endpoint, and each colored line represents a comparable treatment with the probability of being ranked first increasing from the inner to the outer rhombus. Neoadjuvant CRT had the greatest probability of being ranked first among these comparable treatments in terms of all the endpoints. The radar map is depicted according to the Bayesian ranking results based on SUCRA scores presented in supplement. CT, chemotherapy; RT, radiotherapy; CRT, chemoradiotherapy; OS, overall survival; PFS, progression-free survival; DFS, disease-free survival; TFD, reduction in treatment failure due to distant metastasis; TFL, reduction in treatment failure due to locoregional recurrence.

Ranking analysis based on SUCRA scores showed that neoadjuvant CRT with surgery (0.85) was most likely to be the best option in terms of OS benefit. Subsequently, the other treatments were ranked as follows: surgery with adjuvant CRT (0.80), neoadjuvant RT with surgery (0.54), neoadjuvant CT with surgery (0.51), surgery with adjuvant RT (0.40), surgery with adjuvant CT (0.29), and surgery alone (0.11). When different histology types were considered, treatment with the greatest probability of being ranked first was still neoadjuvant CRT. Moreover, neoadjuvant CRT was also most likely to be ranked first for PFS/DFS as well as reducing the rates of locoregional recurrence and distant metastasis. **Figure 4** shows the Bayesian ranking profiles of comparable treatments according to different endpoints (with detailed ranking results summarized in **Supplementary Figure 2**). The ranking of treatments were in line with the pooled analyses using HRs (for OS and PFS/DFS) and ORs (for locoregional recurrence and distant metastasis rates).

### Sensitivity Analysis

To minimize potential bias, sensitivity analysis by the HR calculation method was conducted. When we grouped together studies for which the HRs were obtained by the first two aforementioned approaches, the network meta-analysis showed similar results to primary analysis (**Supplementary Figure 7**).

Besides the analysis based on HR calculation method, the studies by Lee and Mariette were removed from the network due to potential heterogeneous clinical features as previously mentioned. Results did not show deviations compared with the original ones, and neoadjuvant CRT still ruled the entire hierarchy (**Supplementary Figure 8**).

### Study Quality Assessment

**Figure 5:**
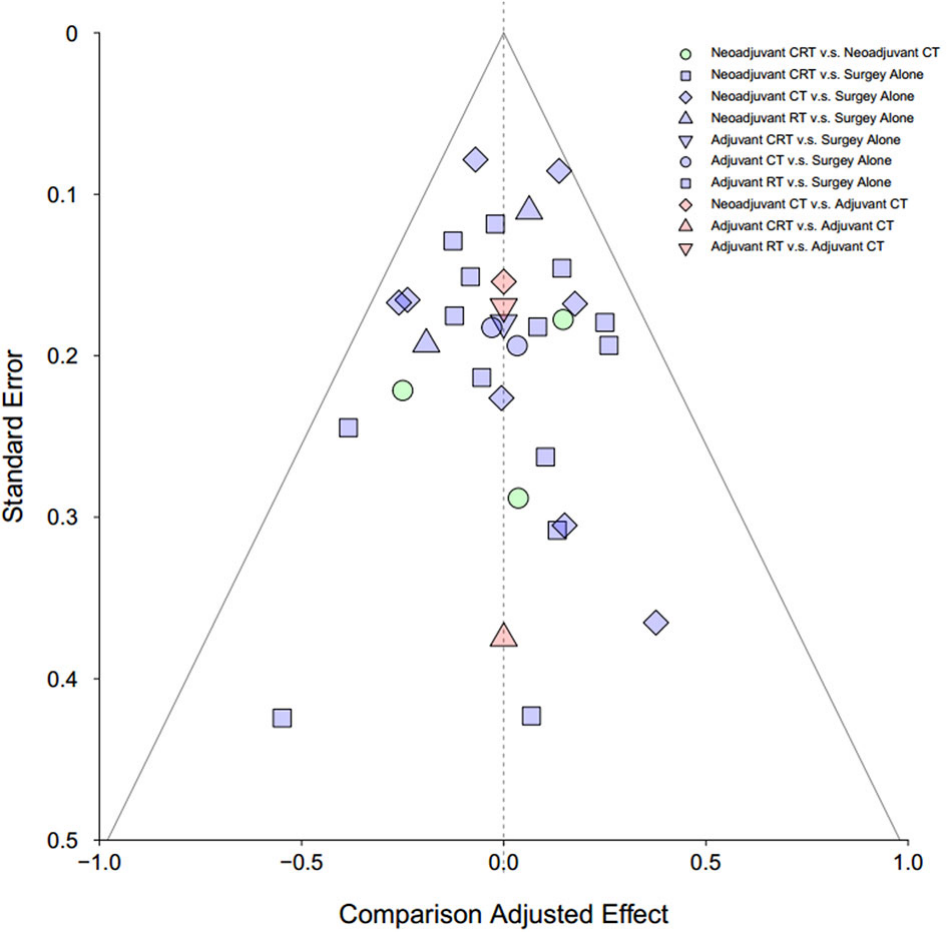
Funnel plot for risk of publication bias in NMA. CT, chemotherapy; RT, radiotherapy; CRT, chemoradiotherapy.

Detailed risk of bias evaluation is given for each study (**Supplementary Figure 4**). There was no eligible RCT deemed at high risk of bias. Due to the nature of treatments, especially the RT, blinding of participants was not possible in clinical settings, and also, the information of blinding of participants was hardly given in the articles. However, we believe that it was unlikely that deviations would arise because of this in the results, and a “low-risk” score was, therefore, given when appropriate. The funnel plot did not indicate any evident risk of publication bias due to the symmetrical distribution (**Figure 5**).

## Discussion

In this NMA of 6168 patients, neoadjuvant CRT followed by surgery is demonstrated to be the most effective approach for resectable esophageal cancer. The results are robust and consistent in several subgroup analyses, including different histology types and statistical methods.

In early exploratory research, due to the limitations of treatment methods, especially RT techniques, the small sample sizes, and the relatively lower quality of studies, many reported that neoadjuvant CRT did not improve patient survival (17, 18, 24), making this treatment model controversial. Over the recent few decades, with RT techniques more advanced, staging work-up more precise and reliable, and systemic therapies with supportive measures improved, the efficacy of neoadjuvant CRT has been identified in more and more high-quality RCTs. The long-term results of the CROSS study confirmed the initially reported survival benefit by neoadjuvant CRT plus surgery compared with surgery alone (6). The most recent NEOCRTEC5010 study added more valuable evidence to the beneficial effect of neoadjuvant CRT over surgery alone (7), whereas phase III trial FFCD 9901 did not demonstrate any beneficial effect between group CRT and group surgery (S) (26). When looking into the inclusion criteria, differences in tumor stages and locations might explain the divergences. The conclusion in our analysis further strengthens the role of neoadjuvant CRT as the preferable treatment modality for patients with resectable esophageal cancer.

There has existed a debate about which treatment is more effective between neoadjuvant CRT and neoadjuvant CT. The conclusions were not consistent among the RCTs. Even though the recent long-term results of the POET study suggested a survival benefit for preoperative CRT compared with preoperative CT (8), von Dobeln and colleagues did not reach the same conclusions as to OS improvement (9). In the latter study, a possible explanation to the lack of survival benefit despite better tumor response could be that significantly more patients treated with neoadjuvant CRT died from postoperative complications. To date, several previous meta-analyses (33, 34) together with our work agree in reporting a proportionally higher OS benefit for neoadjuvant CRT compared with neoadjuvant CT. Moreover, in our NMA, neoadjuvant CRT could improve OS confidently (HR = 0.76, 95% CrI: 0.67–0.85) although the credible interval for neoadjuvant CT was somewhat around the borderline (HR = 0.87, 95% CrI: 0.77–0.98) meaning more uncertainties in neoadjuvant CT.

Several studies suggested that SCC was more sensitive to and carried the potential to benefit even more from current neoadjuvant treatment strategies than AC (6, 9). The results from this NMA also implied that SCC could benefit more from neoadjuvant CRT, which is in accordance with Deng’s and Wang’s meta-analyses (35, 36). Accumulating evidence suggested that esophageal SCC could respond better to CRT than esophageal AC (37). This difference in the responses to CRT of these two subtypes of esophageal cancer may be explained by intrinsic tumor biology between SCC and AC. The difference in tumor biology between SCC and AC may further lead to the different failure patterns with distant metastases being more common in patients with AC (9).

Adjuvant CRT might be recommended for patients receiving surgery without neoadjuvant therapies for certain reasons. The value of adjuvant CRT was confirmed in Kang’s classical meta-analysis (38). In our NMA, although adjuvant CRT apparently demonstrated a similar impact on OS to neoadjuvant CRT, it only exhibited marginal confidence (HR = 0.73, 95% CrI: 0.50–1.09). Only prospective RCTs were included in our study, and therefore, the patients in the adjuvant CRT arm were relatively limited compared with other treatments, and this could partly explain the lack of statistical confidence. Adjuvant therapies may provide a survival benefit for patients with positive lymph nodes or patients with relatively advanced stages in clinical practice. However, this has not been investigated in a formal RCT. Comparatively speaking, when neoadjuvant CRT is available to administer, adjuvant CRT is not believed to be the mainstream option for resectable esophageal cancer.

In our NMA, neoadjuvant CRT not only improved the locoregional control, as confirmed previously, but also reduced distant metastasis. It is partly because patients with higher risk of locoregional recurrence may also have higher risk of distant recurrence (in other words, the probabilities of locoregional and distant recurrence are essentially not statistically independent), and neoadjuvant CRT may reduce distant metastasis by decreasing tumor burden, inhibiting tumor growth, and preventing intraoperative dissemination. Therefore, we believe the improved locoregional control and reduced distant metastasis both contribute to the improved OS by neoadjuvant CRT.

### Limitations

Several considerations should be mentioned when interpreting the results of our NMA. First, even though tests for inconsistency were almost all negative, with the subgroup analyses consistent, the variability in patient populations, treatments, and procedures might not be ignored. Second, we used aggregate data instead of individual patient data, and some unknown or uncontrolled confounding factors may exist. Third, the sample sizes for some treatments were limited: 78 patients included in the adjuvant CRT arm and 150 in the neoadjuvant RT arm. Although adjuvant CRT and neoadjuvant RT ranked second and third in the ranking analysis, even ahead of neoadjuvant CT, some uncertainties might exist, which could also be explained by the wider 95% CrIs, and therefore, the results of ranking analysis should be taken cautiously. Additionally, here we cannot definitively conclude about the optimal CT regimen or RT scheme as the included studies used different agents and radiation dose fractionation. However, a platinum-based CT and a total RT dose of 40–50 Gy as administered in most trials may be reasonable options.

### The Comparison and Strength

Despite these limitations, this NMA aggregated high-quality prospective RCTs of neoadjuvant and adjuvant treatments, summarized and interpreted a wider picture of the evidence base in spite of some head-to-head trials lacking at the moment, and identified the most effective approach through analysis of several different clinical outcomes. The only previously published NMA, conducted by Pasquali in 2016, indicates that neoadjuvant CRT followed by surgery is the only one that could significantly improve OS of patients with resectable esophageal cancer compared with other neoadjuvant or adjuvant treatments (33). However, there exist some early exploratory research that reduce the homogeneity, and the trial of preoperative coupled with postoperative CT was also classified as neoadjuvant CT (39) as the former study was. Besides this, the prescription dose of RT was 20 Gy in some included study (40), which is now believed to be insufficient for neoadjuvant or adjuvant treatments. In this NMA, 4 new or updated RCTs were added, and 6 more loops were generated to expand the evidence base. On the other hand, the studies of lower quality were excluded as described in the inclusion criteria to enhance homogeneity and, therefore, to improve statistical accuracy. PFS/DFS and failure patterns of different treatments were also analyzed to further highlight and confirm our results. Moreover, the calculation method for HRs when they were not available in the original articles was modified and optimized in this NMA.

## Conclusions

This NMA provides evidence that, for patients with resectable esophageal cancer, neoadjuvant CRT and surgery is the optimal strategy in improving the survival, especially for esophageal SCC. Neoajuvant CT might be an appropriate alternative in selected cases. Further studies should focus on the optimization of the most effective neoadjuvant CRT regimens for resectable esophageal cancer.

## Data Availability Statement

The original contributions presented in the study are included in the article/**Supplementary Material**. Further inquiries can be directed to the corresponding author.

## Author Contributions

ZH conceived the study and revised the manuscript. MY, YB, and ZM performed the searches and collected raw data. YM and YW checked the data and performed statistical analysis. MY and YW drafted the manuscript. All authors contributed to the article and approved the submitted version.

## Funding

This study was supported by National Key Research and Development Program (2017YFC1311000).

## Conflict of Interest

The authors declare that the research was conducted in the absence of any commercial or financial relationships that could be construed as a potential conflict of interest.
